# Application value of the treatment of breast cancer bone metastases with radioactive seed 125I implantation under CT-guidance

**DOI:** 10.1186/s12880-021-00726-w

**Published:** 2022-01-04

**Authors:** Haiwen Li, Maobo Wang, Zhenhua Zhu, Yingqiang Lu

**Affiliations:** 1 Department of Radiology, Zibo Hospital of Shandong Guoxin HealthCare Group, Zibo, China; 2 Department of Radiology, Shandong Provincial Maternal and Child Health Care Hospital, No. 238, Jingshi East Road, Jinan City, 250014 Shandong Province China

**Keywords:** CT-guidance, Radioactive seed 125I implantation, Breast cancer, Bone metastases

## Abstract

**Background:**

To investigate the application value of the treatment of breast cancer bone metastases with radioactive seed 125I implantation under CT-guidance.

**Methods:**

A total of 90 patients with breast cancer admitted to our hospital from January 2017 to January 2018 were selected as the research objects and were divided into control group and experimental group according to random grouping, with 45 cases in each group. Conventional treatment was used in the control group, while the treatment of radioactive seed 125I implantation under CT-guidance was used in the experimental group. The clinical efficacy, pain intensity and levels of carcinoembryonic antigen (CEA), carcinoembryonic antigen 153 (CA153), carbohydrate antigen (CA125) in the two groups were compared.

**Results:**

As for the pain intensity, it was evidently lower in the experimental group after treatment than that in the control group (*P* < 0.05); as for the total effective rate, it was obviously higher in the experimental group after treatment than that in the control group (*P* < 0.05); as for the levels of CEA, CA153 and CA125, the data in the experimental group after treatment were much lower than the control group (*P* < 0.05).

**Conclusion:**

Radioactive seed 125I implantation under CT-guidance can effectively improve the effect of the treatment of breast cancer bone metastases. It has curative efficacy and it is worth promoting and using.

## Background

With the increasing pressure of survival and the quickening of pace of life, the incidence rate of breast cancer is increasing. It mostly occurs in middle-aged and elderly people, and it is developing in a younger trend, which seriously endangers people's security of life [1, 2]. In recent years, the treatment of breast cancer can be divided into seed implantation, radiotherapy and chemotherapy, surgical drug therapy and so on. The early treatment methods for breast cancer patients are mostly radiotherapy, chemotherapy and surgical resection. Although effective treatment can be achieved, there are many postoperative complications. Moreover, there are many untoward reactions after radiotherapy and chemotherapy, for example, bone marrow suppression, nausea and vomiting, which affect the quality of life of the patients [2–4]. With the constant improvement of science and technology and the advancement of medical technology, it has gradually become a mainstream topic to further improve the efficacy of treatment and reduce the adverse reactions of patients after treatment, which is also the current research direction of scholars. The radioactive seeds 125I are used for brachytherapy in patients' lesions under CT-guidance. The purpose is to implant the seeds into the patient's lesion and provide uninterrupted treatment to the patient's lesion [5–8].

Radioactive particle therapy can further improve the therapeutic effect, meanwhile, it can kill tumor cells as much as possible and reduce the suffering of patients. At present, radioactive particle therapy has been widely used in major hospitals and achieved significant therapeutic effects [9, 10]. Symptoms can be relieved within 60 days for common solid tumors. According to different conditions, targeted treatment measures can be taken to effectively improve the quality of life of these patients. In order to further explore the application value of the treatment of breast cancer bone metastases with radioactive seed 125I implantation under CT-guidance, 90 cases of breast cancer patients in our hospital from January 2017 to January 2018 are selected as the research objects. The report is summarized as follows:

## Materials and methods

### General information

There are 90 cases of breast cancer patients in our hospital from January 2017 to January 2018 were selected as the research objects. The patients were aged from 40 to 70 years old, with an average age of (51.5 ± 10.9) years old. They were randomly divided into the control group and the experimental group, with 45 cases in each group. The physical data comparison is shown in Table 1.

**Table 1:** Comparison of general information between the two groups ([n(%)]

Classification	RG (n = 45)	CG (n = 45)	X^2^ or t	*P*
Age (years old)	51.5 ± 10.98	51.3 ± 11.01	0.086	0.931
BMI (kg/m^2^)	26.49 ± 1.82	26.56 ± 1.79	0.183	0.854
Previous history				
History of hypertension	21 (46.66)	17 (37.78)	0.728	0.393
History of diabetes	12 (26.67)	15 (33.33)	0.476	0.490
History of chronic bronchitis	12 (26.67)	13 (28.89)	0.055	0.814
Smoking history			0.211	0.645
Yes	2 (4.44)	3 (6.67)		
No	43 (95.56)	42 (93.33)		
Drinking history			0.123	0.725
Yes	4 (8.89)	5 (11.11)		
No	41 (91.11)	40 (88.89)		
Residence			0.052	0.818
Urban	32 (71.11)	31 (68.89)		
Rural	13 (28.89)	14 (31.11)		

### Inclusion criteria

1. Met the diagnostic criteria for breast cancer bone metastasis;
2. Had complete clinical data;
3. The research was approved by the ethics committee of the hospital, and the patients and their family numbers knew the purpose and process of the research and signed the informed consent form.

### Exclusion criteria

1. To combined with malignant tumor;
2. To eliminate physical disability;
3. To eliminate the patients with drug allergy.

### Methods

The control group received conventional treatment with specific measures as follows:

(1) Use strontium chloride (89Sr) injection [Manufacturer: Shanghai Atomic Sinovac Pharmaceutical Co., LTD., the approval number is national medicine permission number H20041312, and the specification is 89 sr 150 MBq (4 mCi)/4 mL (bottle); 89Sr 43.6–90.4 mg/4 mL (bottle)], the dosage is 1.5–2.0 MBq (40–55 μCi)/kg.

Usage: The liquid should be injected slowly and intravenously without dilution. The injections were repeated at least 3 months;

(2) The patients should receive blood examination to ensure that the numeration of leukocyte more than 3500/mm^3^ and blood platelet count more than 80,000/mm^3^.

According to the doctor's advice, regular blood review should be carried out for the patients. If the patient's body has abnormal pain and other symptoms after treatment, the drug dosage can be reduced or targeted treatment can be implemented;

(3) Stop using calcium for more than 2 weeks, the use of drugs must be carried out under the guidance of professional medical personnel.

The experimental group was treated with radioactive seed 125I implantation under CT-guidance. The specific measures were as follows:

1. The patients were checked for various physical indicators before treatment, such as liver function, blood routine, and kidney function;
2. The CT (Somatom Definition AS, Equipment model: Perspective) image was imported into the Treatment Planning System(TPS) to accurately confirm the location of the lesions after scanning, and the activity, number and arrangement of particles required for treatment were calculated according to the drug dose; the diameter of radioactive seed 125I is 0.88 mm, the length is 4.5 mm, and the radiation dose of each seed is 0.4–1.0 mci (average 0.6 mci), half-life is 59.4 d, and it takes 20 d to release 94% of the radiation dose;
3. The patients were given local anesthesia, and the seed implantation needle was placed in the center of the lesion in the patient's body, and parallel needle placement was conducted every 11 mm. Intermittent CT scanning was performed for the lesions, and supplement was carried out for the areas with uneven particle distribution;
4. Monitor the patients' vital signs;
5. If there are complications in the treatment of patients should be dealt with in time.

### Observation index

The indexes of CEA, CA125 and CA153 were observed in the two groups after treatment, and the pain intensity after treatment was compared between the two groups. Using Prism software to make a clear chart for each treatment index.

By comparing the therapeutic effect of the two groups of patients, the evaluation criteria were divided into 4 grades, among which grade I was reduction, fading or disappearance of bone metastasis, and no other new lesions were found; grade II was that the bone metastasis lesions had not been improved and no other new lesions were found; grade III was that bone metastases have improved and new lesions appeared; grade IV was the aggravation or deterioration of bone metastasis lesions, and new lesions appeared. According to the analysis of the treatment results by professionals, the grade I and grade II were judged as effective, grade III and grade IV were judged as invalid, and the total effective rate was the sum of grade I and grade II.

The patients were evaluated by visual analogue Scale (VAS) and told to fill in the form truthfully after treatment. The scale was divided into 10 points, in which 0 point was classified as painless, 1 to 4 was mild pain, 5 to 7 was moderate pain, and 8–10 was severe pain.

### Statistical analysis treatment

The experimental data were statistically analyzed and processed by SPSS21.0 software. The count data was analyzed by x^2^ test and expressed by [n (%)]. The measurement data was analyzed by t-test and expressed by (x ± s). When *p* < 0.05, the difference has statistically significance.

## Results

### Comparison of general information

No significant differences were identified in age, body mass index (BMI), previous history, smoking history, drinking history and residence between the two groups (*P* > 0.05), as shown in Table 1.

### Comparison of pain degree between the two groups

The total pain rate of the experimental group after treatment was obviously lower than that of the control group (*P* < 0.05), as shown in Table 2.

**Table 2:** Comparison of pain degree between the two groups [n(%)]

Groups	n	Painless	Mild	Moderate	Severe	Total pain
RG	45	51.11% (23/45)	35.56% (16/45)	11.11% (5/45)	2.22% (1/45)	48.89% (22/45)
CG	45	24.44% (11/45)	22.22% (10/45)	35.56% (16/45)	17.78% (8/45)	75.56% (34/45)
X^2^						6.806
P						< 0.05

### Comparison of efficacy evaluation between the two groups

The total effective rate of the experimental group was significantly higher than that of the control group (*P* < 0.05), as shown in Table 3.

**Table 3:** Comparison of efficacy evaluation between the two groups

Groups	n	Efficacy evaluation				Total effective rate
		I	II	III	IV	
RG	45	44.44% (20/45)	26.67% (12/45)	17.78% (8/45)	11.11% (5/45)	71.11% (32/45)
CG	45	24.44% (11/45)	17.78% (8/45)	31.11% (14/45)	26.67% (12/45)	42.22% (19/45)
X^2^						7.647
P						< 0.05

### Comparison of CEA levels between the two groups

**Fig. 1:**
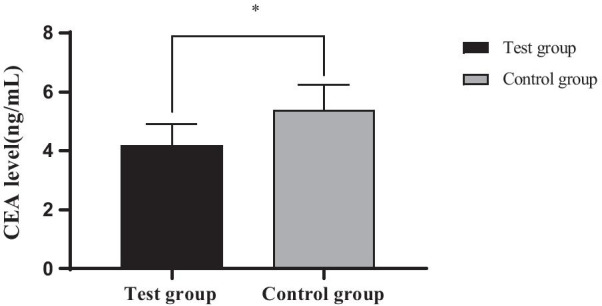
Comparison of CEA levels between the two groups (x ± s). *Note*: The abscissa represents the experimental group and the control group after treatment, and the ordinate represents the CEA level (ng/mL); The CEA level in the experimental group was (3.67 ± 1.03) ng / ml after treatment; The level of CEA in the control group was (4.76 ± 1.23) ng/mL after treatment; *Indicated significant difference in CEA level between the two groups after treatment (t = 4.557, *P* = 0.000)

The CEA level of the experimental group after treatment was significantly lower than that of the control group (*P* < 0.05), as shown in Fig. 1.

### Comparison of CA153 levels between the two groups

**Fig. 2:**
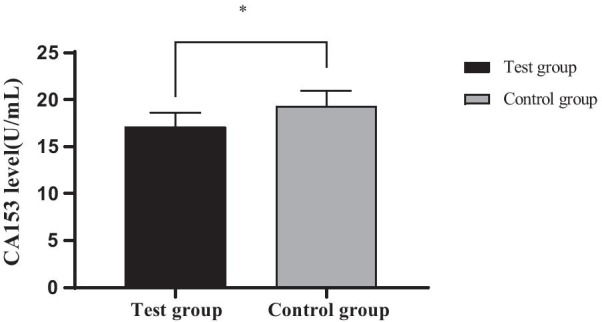
Comparison of CA153 levels between the two groups (x ± s). *Note*: The abscissa represents the experimental group and the control group after treatment, and the ordinate represents the level of CA153, (U/mL); The level of CA153 in the experimental group was (16.1 ± 2.08) U/mL after treatment; The level of CA153 in the control group was (18.14 ± 2.31) U/mL after treatment; *Indicated significant difference in CEA level between the two groups after treatment (t = 4.402, *P* = 0.000)

The CA153 level in the experimental group was obviously lower than that in the control group (*P* < 0.05) after treatment, as shown in Fig. 2.

### Comparison of CA125 levels between the two groups

**Fig. 3:**
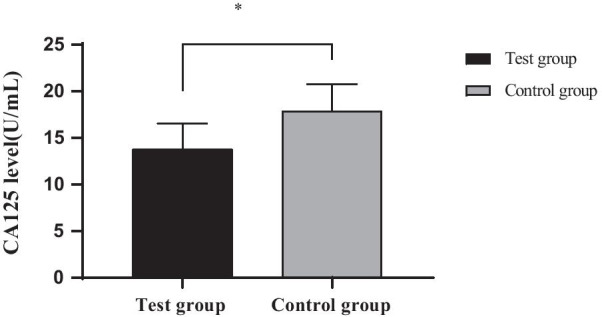
Comparison of CA125 levels between the two groups $\left( {{\overline{\text{x}}} \pm {\text{s}}} \right)$. *Note*: The abscissa represents the experimental group and the control group after treatment, and the ordinate represents the level of CA125, (U/mL); The level of CA125 in the experimental group after treatment was (11.93 ± 3.78) U/mL; The level of CA125 in the control group after treatment was (15.78 ± 4.12) U/mL); *Indicated significant difference in the level of CA125 between the two groups after treatment (t = 4.619, P = 0.000)

The level of CA125 in the experimental group was significantly lower than that in the control group (*P* < 0.05) after treatment, as shown in Fig. 3:

## Discussion

Breast cancer is a malignant tumor occurring in the glandular epithelium of the breast. In recent years, the incidence rate of breast cancer is increasing. The cause of its formation is complex and has not been elucidated yet [11, 12]. The research confirmed that the incidence of the disease is correlated with age. The incidence of the disease is low before the age of 25 and increases gradually after the age of 26. The incidence is high from 49 to 55 years old and decreases gradually after the age of 56. Early diagnosis and treatment can improve the therapeutic effect. Conventional surgical resection of tumors and drug treatment can only relieve part of the patient’s condition, but cannot inhibit the metastasis of cancer cells in time [12–15]. With the continuous improvement and advancement of medical technology, the therapeutic effect of breast cancer has been effectively improved, but there are still some patients with cancer cell proliferation and metastasis after treatment, and bone metastasis, as one of the most common metastasis of breast cancer, has a metastasis rate of up to 71%. Bone metastasis of breast cancer is a complex process, which is mainly manifested as the destruction of bone tissue and release of various growth factors stored in bone tissue through the interaction with bone cells after the metastasis of breast cancer through blood flow to the bone, so that the tumor cells constantly proliferate and form metastasis [16, 17]. Therefore, to seek effective treatment for bone metastasis of breast cancer has become the focus of current research.

This research showed that radioactive seeds 125I implantation therapy under CT-guidance had a significant therapeutic effect on the treatment of breast cancer bone metastases. Radioactive seed implantation can be used to treat the patient’s lesions without interruption according to the TPS plan. After the radioactive seeds are arranged in a fixed order, the seeds are arranged in parallel and evenly distributed [18]. The seeds 125I can be implanted into the tumor with the aid of seed implanter and seed insertion needle, which can continuously release gamma rays and destroy the DNA of tumor cells, so as to inhibit tumor division.

This research showed that the total effective rate of the experimental group after treatment was 71.11%, which was obviously higher than 42.22% of the control group. It is consistent with the research results of Bansal et al. [19]. This article pointed out that “the total effective rate of the combined group after receiving radioactive seed 125I implantation under CT-guidance was 71.43%, which was significantly higher than 45.71% of the control group”, indicating that radioactive seed 125I implantation under CT-guidance is more effective than conventional treatment in the treatment of breast cancer bone metastasis.

## Conclusion

In conclusion, the radioactive seed 125I implantation under CT-guidance can effectively improve the therapeutic effect of breast cancer bone metastases, reduce the pain of patients and improve their quality of life, it has curative effect which is worthy of promotion and use.

## Data Availability

The datasets used and/or analysed during the current study are available from the corresponding author on reasonable request.

## Abbreviations

CEA: Carcinoembryonic antigen
TPS: Treatment planning system
